# Stabilizing Buried Interface via Synergistic Effect of Fluorine and Sulfonyl Functional Groups Toward Efficient and Stable Perovskite Solar Cells

**DOI:** 10.1007/s40820-022-00992-5

**Published:** 2022-12-29

**Authors:** Cheng Gong, Cong Zhang, Qixin Zhuang, Haiyun Li, Hua Yang, Jiangzhao Chen, Zhigang Zang

**Affiliations:** 1 Key Laboratory of Optoelectronic Technology and Systems (Ministry of Education), Chongqing University, Chongqing, 400044 People’s Republic of China; 2 Institute of High Energy Physics, Chinese Academy of Sciences (CAS), Beijing, 100049 People’s Republic of China

**Keywords:** Perovskite solar cells, Buried interface, Multiple chemical bonds, Synergistic effect of functional groups, Defect passivation

## Abstract

The interfacial defects and energy barrier are main reasons for interfacial nonradiative recombination. In addition, poor perovskite crystallization and incomplete conversion of PbI_2_ to perovskite restrict further enhancement of the photovoltaic performance of the devices using sequential deposition. Herein, a buried interface stabilization strategy that relies on the synergy of fluorine (F) and sulfonyl (S=O) functional groups is proposed. A series of potassium salts containing halide and non-halogen anions are employed to modify SnO_2_/perovskite buried interface. Multiple chemical bonds including hydrogen bond, coordination bond and ionic bond are realized, which strengthens interfacial contact and defect passivation effect. The chemical interaction between modification molecules and perovskite along with SnO_2_ heightens incessantly as the number of S=O and F augments. The chemical interaction strength between modifiers and perovskite as well as SnO_2_ gradually increases with the increase in the number of S=O and F. The defect passivation effect is positively correlated with the chemical interaction strength. The crystallization kinetics is regulated through the compromise between chemical interaction strength and wettability of substrates. Compared with Cl^−^, all non-halogen anions perform better in crystallization optimization, energy band regulation and defect passivation. The device with potassium bis (fluorosulfonyl) imide achieves a tempting efficiency of 24.17%.

**Supplementary Information:**

The online version contains supplementary material available at 10.1007/s40820-022-00992-5.

## Highlights

- An effective buried interface stabilization strategy based on synergistic effect of fluorine and sulfonyl functional groups is proposed.
- The correlations between molecular structures, defect passivation, interfacial energy band alignment, perovskite crystallization and device performance are established.
- The device with KFSI achieves an impressive efficiency of 24.17%.

**Supplementary Information:**

The online version contains supplementary material available at 10.1007/s40820-022-00992-5.

## Graphical abstract

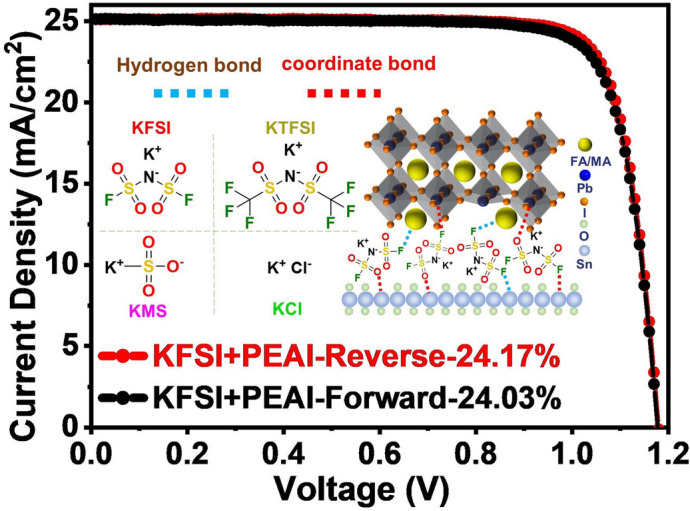

## Introduction

These merits including low cost, solution processing and outstanding power conversion efficiency (PCE) make perovskite solar cells (PSCs) attract mammoth attention in academia and industry [1–5]. The superior properties of perovskites should be responsible for the extremely rapid PCE increase from 3.8% in 2009 [6] to presently certified 25.7% [7]. However, the further performance improvement is severely limited by the interfacial nonradiative recombination [8]. In regular PSCs, the interface of electron transport layer (ETL) with perovskite layer is usually referred to as buried interface. It is very difficult to perform in situ characterization of the bottom surface of perovskite films [9]. Therefore, it is much more challenging to modify buried interface compared with the perovskite/hole transport layer (HTL) interface. Buried interfacial defects are a main reason of interfacial non-radiative recombination [10]. The charge trap density at grain boundary (GB) and interface was reported to be much larger than that within perovskite grains [11]. Moreover, the donor type defects and acceptor type defects may exist simultaneously at buried interface which are usually deep-level defects [12, 13]. It is well known that considerable quantity of Sn interstitial defects (Sn_i_) and oxygen vacancy defects (V_o_) usually distribute at the surface and in the interior of SnO_2_ ETL [14]. Sn_i_ and V_o_ defects can be formed spontaneously because of their low formation energies which can affect the photoelectric properties and energy levels of SnO_2_ [15]. The trap carriers at the heterojunction interface are easy to be trapped by interfacial deep-level defects, resulting in interfacial non-radiative recombination losses and accordingly diminishing PCE and stability. Apart from buried interface defects, interfacial energy barrier resulting from imperfect energy band alignment also could result in interfacial carrier nonradiative recombination [8]. Interface modification by appropriate materials can improve interfacial energy band alignment and minimize interfacial energy barrier, resulting in enhanced device performance [12, 16, 17]. The quality of perovskite films plays a key role in fabricating stable and efficient PSCs. The perovskite film quality is primarily determined by its crystallization process. One step and two step approaches are usually employed to prepare perovskite films. Nevertheless, the PCEs of the PSCs based on two step method [18–20] are still lower than that of the PSCs using one step method [4, 21]. This should be due to more difficult crystallization control for two step method as compared to one step method. It has been extensively demonstrated that it is an effective approach to modulate perovskite crystallization by modifying ETL substrates [22, 23]. In a word, multifunctional molecules are urgently needed to be developed to manage interfacial carrier through simultaneous realization of interfacial defects passivation, interfacial energy band alignment optimization and perovskite crystallization modulation.

To date, huge efforts have been devoted to developing various materials to modify buried interface. However, reported most interface molecules have relatively simple functions, either passivating defects, tuning interfacial energy band alignment or modulating perovskite crystallization [24–26]. It is of great importance for simultaneously achieving multiple functions to enrich chemical bonding modes (e.g., ionic bond, coordination bond and hydrogen bond). Among various interface materials, salts containing both cation and anion are the most appropriate candidate for realizing multiple chemical bonds with perovskite and SnO_2_ layers [16, 17, 27, 28]. The anions and cations in salts can form ionic bonds with charged defects in perovskite films and thus passivate simultaneously positively and negatively charged defects. Fluorine functional groups incorporating into cations and/or anions can not only form hydrogen bond with organic cations in perovskites but also form coordination bond with Pb^2+^ and Sn^4+^ [17, 28]. This suggests that fluorination strategy is a feasible and effective approach to accomplish multiple functions induced by multiple chemical bonds. Except for fluorination strategy, introduction of functional groups (e.g., C=O [29, 30], S=O [16], and C=S [31, 32]) in cation and anion is another effective method for enriching chemical interaction modes because these ligand functional groups can effectively passivate defects and control crystallization kinetics. In recent several years, the additive or interface molecules containing non-halogen anions have received considerable attention [33], Non-halogen anions play an important role in defect passivation (HCOO^−^, CO_3_^2−^, PO_4_^3−^ and NO_3_^−^) [34–37], crystallization regulation (HCOO^−^, SCN^−^ and SO_4_^2−^) [2, 37–41], and energy level alignment modulation (BF_4_^−^ and PF_6_^−^) [17, 28, 42]. However, compared with halide anions, the working mechanism of non-halogen anions is still obscure. In addition, the synergistic effect of non-halogen and halogen functional groups in same anions is still not revealed up to now. Here, it needs to be noted that most researches often adopted single non-halogen anion to modify buried interface [28, 39]. For example, the use of a single non-halogen anion to improve crystallinity and interfacial carrier transport in perovskite films has been reported by Singh et al. and Chen et al. [43, 44]. The specific roles of various functional groups contained in non-halogen anions and the laws of their synergistic effects have not yet been revealed. At the same time, it is difficult for non-halogen anions to be used at the perovskite/HTL interface to exert their maximum potential, because the modified molecules can only interact with the perovskite film and not with the HTL layer. However, this is not beneficial for maximizing the potentials of non-halogen anions. Therefore, it is urgently needed to systematically and deeply uncover the relationships between structures of non-halogen anions, properties of non-halogen anions, defect density, interfacial carrier dynamics and device performance.

In this work, we proposed a buried interface stabilization strategy based on synergistic engineering of fluorine and sulfonyl functional groups, which enhanced the PCE and stability of planar PSCs using two step method. Potassium chloride (KCl, reference molecule), potassium methanesulfonyl (KMS), potassium bis (fluorosulfonyl) imide (KFSI) and potassium bis (trifluoromethanesulfonyl) imide (KTFSI) were used to modulate buried interface. S=O functional group and non-halogen anion can form coordination bond with Pb^2+^ and Sn^4+^. K^+^ and non-halogen anions can form ionic bonds with cation and halide vacancy defects, respectively. In short, multiple chemical bonds between modifiers and functional layers were achieved. The defect passivation effect was determined by chemical interaction strength. For energy band modulation, appropriate number of S=O and F functional groups was required. In case of crystallization control, the compromise between chemical interaction strength and wettability of substrates should be considered simultaneously. The introduction of F would enhance chemical interaction while it also would increase hydrophobicity of surface of ETL. Appropriate hydrophobicity is conducive to inhibiting heterogeneous nucleation and thus facilitating perovskite crystallization but too high hydrophobicity hinders perovskite crystallization. Taken together, KFSI is the best in increasing device performance. Compared with Cl^−^, all non-halogen anions exhibited better passivation effect, energy band alignment and perovskite crystallization, which highlights importance of non-halogen anions in interface engineering. The KFSI-modified device delivered a promising PCE of 24.17% along with excellent stability. This work demonstrated superiority of non-halogen anions and provide a guidance for enhancing photovoltaic performance of PSCs by managing interfacial carrier via the synergistic design of organic functional groups.

## Experimental Section

### Materials

The tin (IV) oxide (SnO_2_, 15% in H_2_O colloidal dispersion) was bought from Alfa Aesar. Lead (II) iodide (PbI_2_, 99.99%), Spiro-OMeTAD (99.86%), formamidine hydroiodide (FAI, 99.5%), methylammonium iodide (MAI, 99.9%), and methylamine hydrochloride (MACl, 99.9%) were bought from Advanced Election Technology Co., Ltd. N, N-dimethylformamide (DMF, 99.8%), 2-propanol (IPA, 99.9%), dimethyl sulfoxide (DMSO, 99.9%), chlorobenzene (CB, 99.8%), and acetonitrile (ACN, 99.8%) were obtained from Sigma-Aldrich. Bis(trifluoromethane) sulfonimide lithium salt (Li-TFSI, 99%) and 4-tertbutylpyridine (tBP, 99%) were obtained from Xi’an Polymer Light Technology Corp. Potassium chloride (KCl, 99.8%), potassium methanesulfonate (KMS, 98.0%), potassium bis (trifluoromethanesulfonyl) imide (KTFSI, 97%), potassium bis (fluorosulfonyl) imide (KFSI, 97%) were purchased from Aladdin.

### Device Fabrication

The etched ITO glass was ultrasonically cleaned sequentially by detergent, deionized water, ethanol, acetone and IPA. The SnO_2_ colloidal solution was spin-coated on the ITO substrates at 4000 rpm for 30 s and then the SnO_2_-coated ITO substrates were annealed at 150 °C for 30 min. Afterwards, SnO_2_ films were treated by UV-ozone for 15 min. For modified SnO_2_ films, different concentrations of KCl solution in water, KMS solution in IPA, KTFSI solution in IPA and KFSI solution in IPA were spin-coated onto the SnO_2_ films at 5000 rpm for 30 s and annealed at 100 °C for 5 min. The PbI_2_ solution (691.5 mg, 1.5 mmol mL^−1^) in DMF:DMSO (9:1) was spin-coated onto pristine and modified SnO_2_ films at 1500 rpm for 30 s and then PbI_2_ films were annealed at 68 °C for 1 min. After the PbI_2_ film cooled down to room temperature, 45 μL of organic mixture solution of FAI (90 mg), MAI (6.39 mg) and MACl (9 mg) in 1 mL IPA was spin-coated onto PbI_2_ films at 2300 rpm for 30 s, and then the films were transferred to ambient air condition (30–40% humidity) and annealed at 150 °C for 15 min. For PEAI modified perovskite films, the 2 mg mL^−1^ of PEAI solution in IPA was spin-coated onto the perovskite films at 5000 rpm for 30 s without annealing. The hole transport material solution was prepared through dissolving 72.3 mg Spiro-OMeTAD, 35 μL Li-TFSI stock solution (260 mg Li-TFSI in 1 mL acetonitrile), and 30 μL tBP in 1 mL CB. Then the hole transport layer (HTL) was prepared by spin-coating a Spiro-OMeTAD solution on the top of the perovskite layer at 4000 rpm for 30 s. Finally, 100 nm of Ag electrode was thermally evaporated on HTL using a shadow mask.

### Film Characterization

The field emission scanning electron microscopy (FE-SEM, JEOL JSM-7800F) was applied to characterize cross-sectional and surface morphology of perovskite films. X-ray diffraction (XRD) patterns were acquired using a PANalytical Empyrean diffractometer equipped with Cu Kα radiation (*λ* = 1.5406 Å). X-ray photoelectron spectroscopy (XPS) and ultraviolet photoelectron spectroscopy (UPS) were measured by Thermo Fisher Escalab 250Xi spectrometer using a monochromatized Al source. XPS was calibrated using the peak position of C 1*s* and UPS was calibrated using the work function of Au. In particular, the samples tested for XPS measurements were prepared by spin-coating the perovskite precursor solutions with different modifiers and annealed to form perovskite films. The optical absorption and transmission spectra were measured by Shimadzu UV3600 Spectrophotometer. The steady-state and time-resolved photoluminescence spectra were performed with a fluorescence spectrophotometer (FLS1000, Edinburgh Instruments Ltd.) which was equipped with a pulse laser diode with a wavelength of 450 nm. The conductivity of SnO_2_ were carried out on Keithley 2400 source meter with a structure of ITO/PCBM/SnO_2_ without or with KCl, KMS, KFSI and KTFSI/perovskite/PCBM/Ag. Two-dimensional grazing-incidence wide-angle X-ray scattering (GIWAXS) images were collected on BL1W1A at the Beijing Synchrotron Radiation Facility (BSRF) (*λ* = 1.54 Å). The trap state density (*n*_t_) was determined by the onset of the trap filling limit voltage (*V*_TFL_) according to Eq. (1) [45]:

1 $$V_{{{\text{TFL}}}} = ({\text{en}}_{{\text{t}}} L^{{2}} )/(2\varepsilon_{{0}} \varepsilon_{{\text{r}}} )$$

where *ε*_0_ is the permittivity of free space, *ε*_r_ is the relative permittivity of perovskite, *e* denotes the elementary charge and *L* is the thickness of perovskite layer. Space charge limited current (SCLC) measurement was applied to determine the electron trap density and mobility using the electron-only device with a structure of ITO/SnO_2_/(KCl, KMS, KFSI or KTFSI)/PCBM/Ag. The SCLC method was employed to measure the electron mobility of the pristine SnO_2_ film and modified SnO_2_ films. The electron mobility (*μ*_e_) is calculated by Eq. (2) [46]:

2 $$\mu_{{\text{e}}} = \frac{{8{\text{JL}}^{3} }}{{9\varepsilon \varepsilon_{0} (V_{{{\text{app}}}} - V_{{{\text{bi}}}} )^{2} }}$$

where *J* is the current density, *L* is the thickness of ETL, *ε* is the relative dielectric constant of ETL, *ε*_0_ is the vacuum permittivity, *V*_app_ is the applied voltage, and *V*_bi_ is the built-in voltage due to the different work function of the two electrodes. The Fourier transforms infrared (FTIR) spectra were recorded by Nicolet iS50 Infrared Fourier transform microscope (Thermo Fisher Scientific) in the transmittance mode. The samples with a structure of ITO/SnO_2_/without or with KCl, KMS, KFSI or KTFSI/perovskite film were prepared for FTIR measurements. The texture coefficient (*TC*) of the specified plane is determined from the XRD spectra and calculated by Eq. (3) [47],

3 $${\text{TC}}\left( {{\text{hkl}}} \right) = \left\{ {\frac{{I_{{\text{m}}} \left( {{\text{hkl}}} \right)}}{{I_{{0}} ({\text{hkl}})}}} \right\}\left\{ {\frac{1}{{\text{n}}}\mathop \sum \limits_{{1}}^{{\text{n}}} \frac{{I_{{\text{m}}} \left( {{\text{hkl}}} \right)}}{{I_{{0}} ({\text{hkl}})}}} \right\}^{ - 1}$$

where *I*_m_(*hkl*) is intensity of the (*hkl*) diffraction peak on the sample under investigation, *I*_0_(*hkl*) is the intensity of the (*hkl*) plane from a powder diffraction file, and *n* is the number of diffractions considered in the analysis. The powder diffraction profiles of FAPbI_3_ for reference were taken from the report of Stoumpos et al. [48].

### Device Characterization

The current density–voltage (*J-V*) characteristics of the devices were measured in ambient air (the relative humidity was 40%–50%) by a solar simulator equipped with 450 W Xenon lamp (Newport, 2612A) and a Keithley 2400 source meter. Light intensity was adjusted to AM 1.5G one sun (100 mW cm^−2^) with a NIM calibrated standard Si solar cell. A metal mask with an aperture area of 0.04 cm^2^ was applied on top of the cell to define active area. The incident photon-to-current efficiency (IPCE) measurement was performed on a Newport Instruments system (Newport-74125) coupled with a lock-in amplifier and a 300 W Xenon lamp. Transient photocurrent (TPC) and transient photovoltage (TPV) measurements were performed using a system excited by a 532 nm (1000 Hz, 6 ns) pulsed laser. Recording photocurrent or photovoltage decay process used a 1 GHz Agilent digital oscilloscope (DSO-X3102A) with a 50 X or 1 MX sampling resistor. The device stacks used to measure TPC and TPV are complete device, which includes the substrate ITO, electron and hole transport layer, and silver electrode.

## Results and Discussion

### Chemical Interaction Mechanisms Investigation

**Fig. 1:**
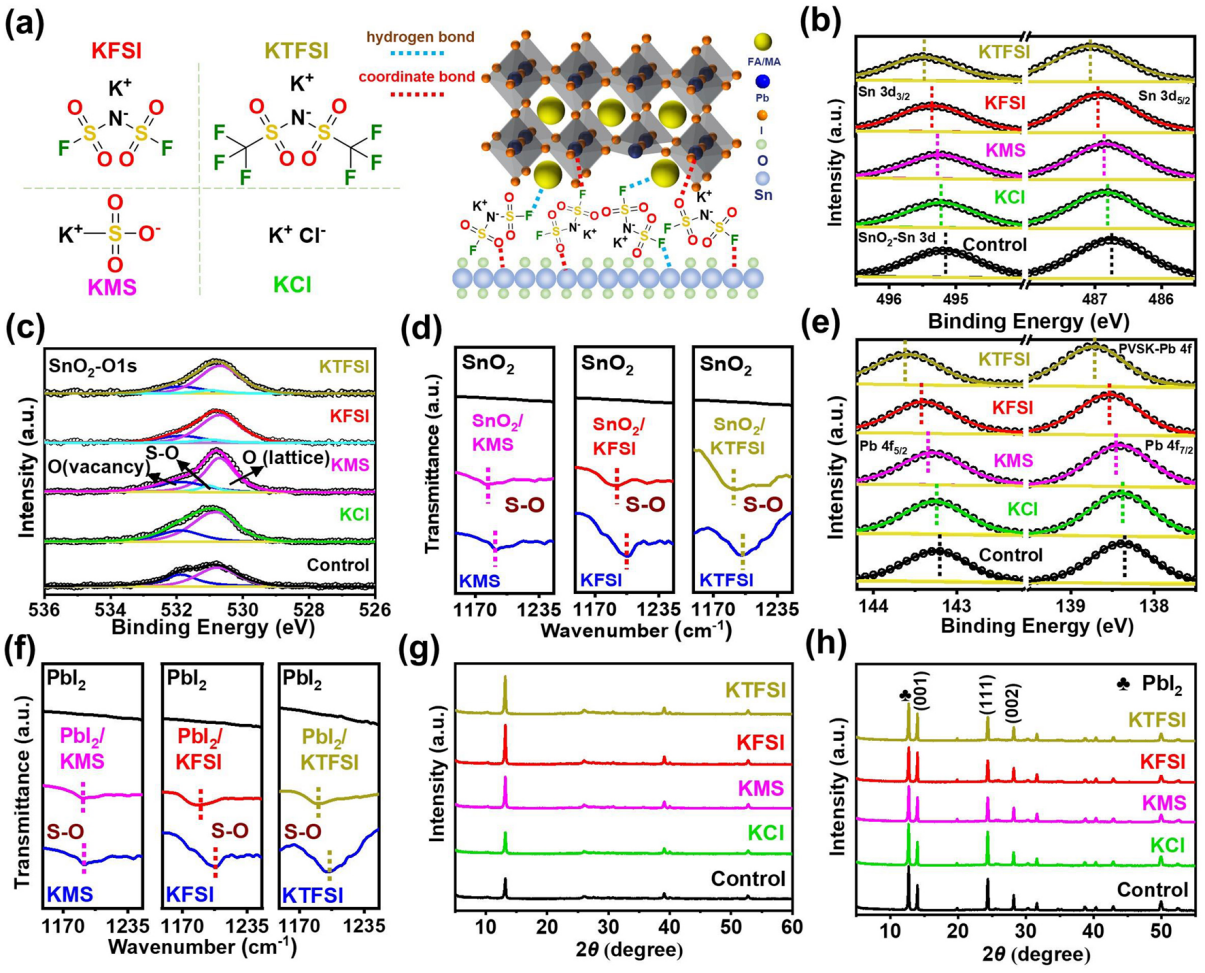
**a** Molecular structures of potassium salts used for modifying buried interface and schematic illustration of the interaction of modifiers and functional layers. **b** Sn 3d and **c** O 1*s* XPS spectra of the control and modified SnO_2_ layers. **d** FTIR spectra of pure KMS, KTFSI and KFSI and the SnO_2_ films without and with KMS, KTFSI and KFSI. **e** XPS spectra of the control and modified perovskite films. **f** FTIR spectra of the PbI_2_ films with or without KMS, KTFSI and KFSI. XRD patterns of the **g** PbI_2_ films and **h** perovskite films deposited on SnO_2_ ETLs without or with KCl, KMS, KTFSI and KFSI

Figure 1a shows the molecular structures of KCl, KMS, KFSI and KTFSI. It is schematically illustrated that modification molecules could bridge perovskite and SnO_2_ films by rich chemical bonds. The chemical interactions between modifier and perovskites or ETL were investigated by XPS and FTIR spectroscopy measurements. In Fig. 1b, the binding energies of Sn 3d_5/2_ and Sn 3d_3/2_ of the pristine SnO_2_ layer were increased inch by inch in the order of KCl, KMS, KFSI and KTFSI. Among them, the sample modified by KTFSI had the largest binding energy shift, which indicates that the interaction of modifiers with ETL is positively correlated with the quantity of F and S=O. It is found from Fig. 1c that O 1*s* peak can be divided into two peaks. One is lattice oxygen (O_L_) and the other is vacancy oxygen (O_V_) [12, 28]. The KMS, KFSI and KTFSI-modified SnO_2_ had a characteristic peak of S–O, and the enlarged view is shown in Fig. S1. The peak area ratios of O_V_ to O_L_ were calculated to be 0.66, 0.65, 0.42, 0.38 and 0.29 for bare, KCl, KMS, KFSI and KTFSI modified SnO_2_, respectively. This shows that passivation effect of anions for oxygen vacancy defects gradually improved with the increase in chemical interaction strength. SnO_2_-KTFSI had the smallest oxygen vacancy density, suggesting that the incorporation of strongly coordinated F and S=O functional groups is favorable for defect passivation. In Fig. S2, the Sn–O peak in bare SnO_2_ sample was at 695 cm^−1^, which was moved to 698, 705, 709 and 714 cm^−1^ for KCl, KMS, KFSI and KTFSI treated SnO_2_, respectively. This indicates that all anions can interact with SnO_2_ but stronger interaction was found for non-halogen anions than Cl^−^, which could be presence of F and S=O in non-halogen anions. Interestingly, a new peak attributed to Sn-F bond appeared in SnO_2_-KTFSI and SnO_2_-KFSI, implying that F in FSI^−^ and TFSI^−^ can coordinate with undercoordinated Sn^4+^ [17, 28]. In Fig. 1d, the peaks around 1191.1, 1203.9 and 1198.1 cm^−1^ belonging to S=O stretching vibration in pure KMS, KFSI and KTFSI, respectively, whereas the peaks were shifted to 1185.4, 1195.6 and 1186.6 eV for KMS, KFSI and KTFSI modified SnO_2_ films, respectively, which confirmed the interaction between S=O and undercoordinated Sn^4+^ and/or oxygen vacancies. Likewise, peak shift degree gradually increased in the order of KMS, KFSI and KTFSI. Figure 1e reveals that the binding energies of Pb 4f_5/2_ and Pb 4f_7/2_ were gradually increased from the control film (143.24 and 138.26 eV), the perovskite film with KCl (143.26 and 138.38 eV), the perovskite film with KMS (143.29 and 138.41 eV), the perovskite film with KFSI (143.40 and 138.52 eV) to the perovskite film with KTFSI (143.58 and 138.70 eV), which should be due to interaction of Pb^2+^ with S=O and/or F. Moreover, the S–O peak shift also revealed the interaction between S=O and Pb^2+^ (Fig. 1f). We found in Fig. S3 that N–H peak was not shifted for KCl and KMS modified perovskite films with respect to the control film while the peak shift degree gradually increased from KFSI to KTFSI, which is indicative of gradually enhanced hydrogen bond strength with the increase in the number of F functional group. N 1*s* XPS spectra in Fig. S4 and FTIR spectra in Fig. S5 also certified the above change tendency of hydrogen bond strength. In conclusion, we have demonstrated that anions in modifiers can chemically interact with both ETL and perovskites and chemically link the two important functional layers. Furthermore, it was uncovered that F and S=O in non-halogen anions can interact with both functional layers. The strong chemical interaction should contribute to improved interfacial contact and thus enhancing PCE and stability.

Figures S6 and S7 demonstrate that light transmittance and bandgap of SnO_2_ almost did not change after the modification of different potassium salts. After modification, the conductivity and electron mobility of ETL was slightly increased (Figs. S8 and S9), which is attributed to effective passivation of these modifiers for oxygen vacancy and undercoordinated Sn^4+^ defects [49]. The UPS test was performed to calculate energy band values of the control and modified ETLs (Fig. S10). It is seen from Fig. S11 and Table S1 that the conduction band minimum (CBM) and valence band maximum (VBM) were gradually upshifted in the order of bare SnO_2_, SnO_2_-KCl, SnO_2_-KMS, SnO_2_-KTFSI and SnO_2_-KFSI. The introduction of highly electronegative F and S = O in non-halogen anions accounts for upshifted CBM and VBM. Improved interfacial energy band alignment should promote electron extraction and transfer, which leads to inhibiting interfacial nonradiative recombination and increasing photovoltaic parameters.

### Crystallization and Morphology of Perovskite Films

XRD measurement was performed to study the crystal structure and crystallinity of the PbI_2_ and perovskite films without and with KCl, KMS, KFSI and KTFSI. As presented in Fig. 1g, the characteristic diffraction peak intensity of PbI_2_ was gradually increased from pristine PbI_2_, PbI_2_-KCl, PbI_2_-KMS, PbI_2_-KTFSI to PbI_2_-KFSI, suggesting that these modifiers can accelerate the crystallization of PbI_2_ films. From Fig. 1h, the same change trend was observed for the perovskite films. The peak intensity of PbI_2_ gradually reduced and the intensity of (001) crystal plane from perovskites gradually increased in the order of the control perovskite, perovskite with KCl, perovskite with KMS, perovskite with KTFSI and perovskite with KFSI. The gradually enhanced *TC* in Table S2 and Fig. S12 indicates that the perovskite grew along (001) crystal plane after interface modification and the orientation growth was gradually enhanced in the order of KCl, KMS, KTFSI and KFSI-modified perovskite films. (001) crystal plane is perpendicular to the substrate and is can facilitate carrier transport and collection. The strong chemical interaction is beneficial for the crystallization of PbI_2_ and perovskite films but superabundant F would increase contact angle and reduce wettability of ETL substrates (Fig. S13). It was reported that appropriate hydrophobicity is conducive to repressing heterogeneous nucleation and promoting perovskite homogeneous nucleation [49]. Consequently, the KFSI-modified perovskite film exhibited higher crystallinity compared with KTFSI-modified one. It shows that interfacial chemical interaction and wettability should be simultaneously considered when we design interface molecules.

**Fig. 2:**
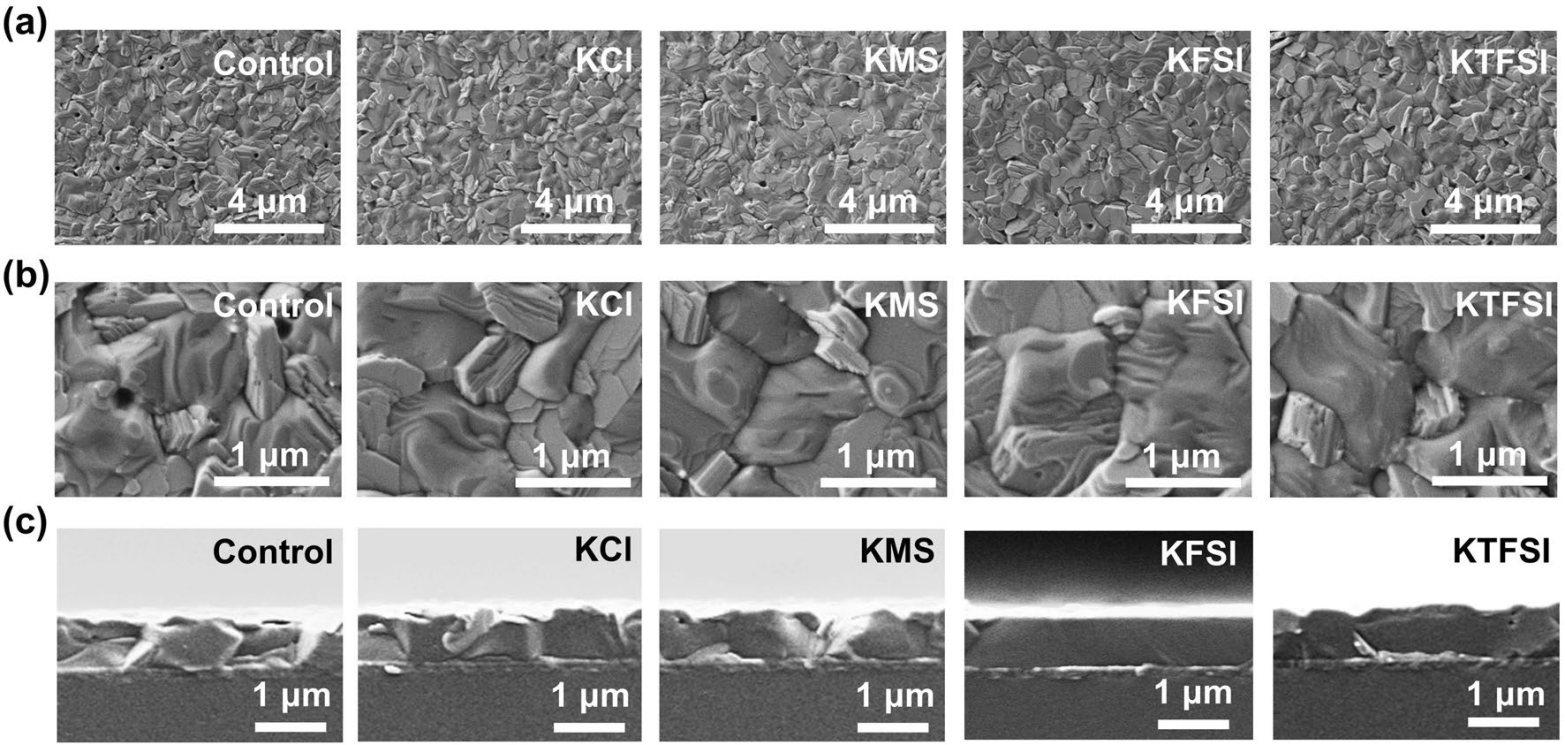
**a, b** Top-view and **c** cross-sectional SEM images of the control and modified perovskite films

The SEM images of pristine PbI_2_ and PbI_2_ films modified by KCl, KMS, KFSI or KTFSI are shown in Fig. S14. The KFSI-modified PbI_2_ films show uniform and dense morphology, which could be beneficial to form smooth and highly crystalline perovskite layer [50]. As shown in Fig. S15, the bright rings at *q*_xy_ = 0.9 and 1.0 Å^−1^ (where *q*_xy_ was the scattering vector, *q*_xy_ = 4π sin*θ*/*λ*) were the diffraction peaks for (001) crystal planes of PbI_2_ and perovskite, respectively [51]. The diffraction spot of perovskite corresponding to (001) crystal plane became more and more discrete in the order of control, KCl-, KMS-, KTFSI- and KFSI-modified perovskite films. This suggests that the conversion of PbI_2_ to perovskite was promoted and the perovskite crystallization was improved after interface medication. Moreover, non-halogen anions were more effective in modulating the crystallization in contrast to Cl^−^. The synergistic effect of F and S=O functional groups play a key role in preparing superior perovskite layers. The SEM images of the perovskite films based on the bare and modified SnO_2_ substrates are exhibited in Fig. 2a-b. The uniformity and compactness were improved after interface modification. The grain size was increased from 0.76 μm of the pristine perovskite film to 0.82, 0.93, 1.14 and 1.03 μm of the films modified by KCl, KMS, KFSI and KTFSI, respectively (Fig. S16). The enlarged grain size and ameliorated morphology are attributed to the improved crystallization. We can see from Fig. 2c that the cross section of the control perovskite film was composed of small perovskite grains while the large perovskite grain was across the whole cross section for modified perovskite films. Moreover, the KFSI modified perovskite film seems to show the best cross section, which is in good agreement with its highest crystallinity and best orientation revealed by XRD and GIWAXS results.

### Carrier Lifetimes and Defect Densities of Perovskite Films

**Fig. 3:**
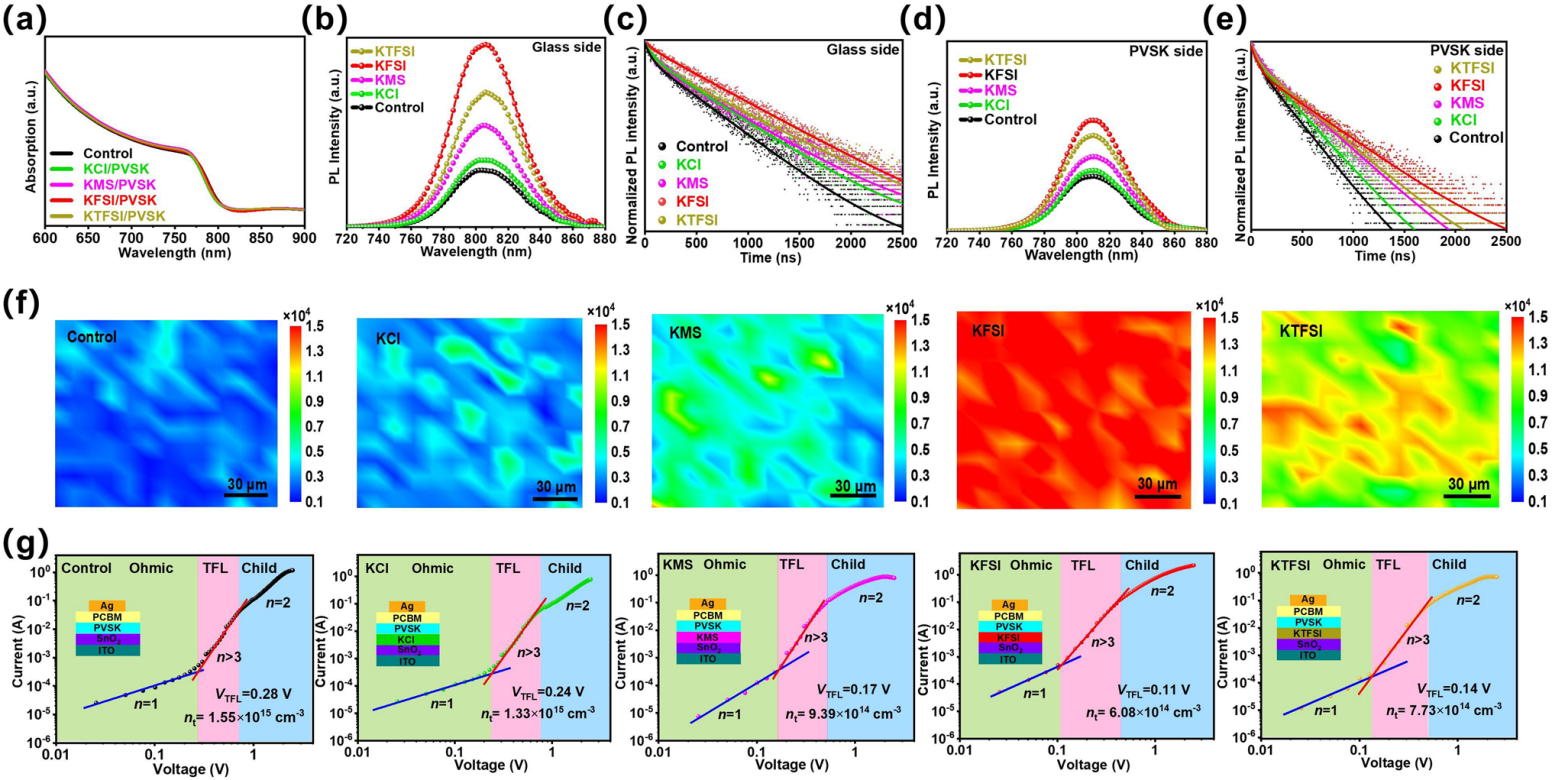
**a** UV–vis absorption spectra of the perovskite films with or without modifiers. **b, d** SSPL and **c, e** TRPL spectra of the perovskite films prepared on non-conductive glass without and with modifiers by measuring from the **b, c** glass side or **d, e** perovskite side. **f** PL mapping images of the perovskite films deposited on non-conductive glass without and with modifiers. **g** Dark *I–V* curves of the electron-only devices

We investigated the light harvesting properties of the perovskite films without and with modifiers by performing ultraviolet–visible (UV–vis) absorption measurement. As exhibited in Fig. 3a, similar absorption properties were found for all perovskite films. All potassium salts modification can increase photoluminescence (PL) intensity regardless of measuring direction (perovskite or glass side). However, we observed the more significant PL intensity enhancement upon detecting from glass side as compared to perovskite side, indicating modifier could effectually cure buried interface (Fig. 3b-d). The time-resolved PL (TRPL) curves were fitted by the double exponential function Eq. (4):

4 $$I(t) = I_{0} + A_{1} e^{{( - t/\tau_{1} )}} + A_{2} e^{{( - t/\tau_{2} )}}$$

where *A*_1_ and *A*_2_ represent the decay amplitude of fast and slow decay processes, respectively, *τ*_1_ and *τ*_2_ stand for the fast and slow decay time constants, respectively [28, 52]. The average carrier lifetime (*τ*_ave_) is obtained through using Eq. (5):

5 $$\tau_{{{\text{ave}}}} = (A_{1} \tau_{1}^{2} + A_{2} \tau_{2}^{2} )/(A_{1} \tau_{1} + A_{2} \tau_{2} )$$

The corresponding fitting data are displayed in Table S3. The carrier lifetime change tendency from perovskite side is same as that from glass side but enhancement degree of the latter is much larger than that of the former, which agrees with steady state PL (SSPL) results. It was shown that non-halogen anions possessed better defect passivation effect relative to Cl^−^. The strong chemical interaction is conducive to passivating defects but far too many F would remarkably increase the hydrophobicity of surface of ETLs and thus is not beneficial for perovskite crystallization. The reproducibility of the above PL results was affirmed by PL mapping images in Fig. 1f.

The trap density of the perovskite films without and with modifiers was quantified by the space charge limited current (SCLC) measurement in Fig. 3k. The perovskite film with KFSI possessed the least trap density of 6.08 × 10^14^ cm^−3^, which was increased to 7.73 × 10^14^, 9.39 × 10^14^, 1.33 × 10^15^ and 1.55 × 10^15^ cm^−3^ for the KTFSI, KMS, KCl and control films, respectively. This suggests that the defect passivation effect increased in the order of KCl, KMS, KTFSI and KFSI. Reduced defect density and increased carrier lifetimes are ascribed to effective defect passivation and improved perovskite crystallization. Although KTFSI had the best defect passivation effect due to its strongest interaction with perovskite as well as ETL, KFSI had the smallest defect density and longest carrier lifetime, which is because of the trade-off between defect passivation effect and crystallization of PbI_2_ and perovskite. It can be concluded that appropriate number of F is of great importance for simultaneously considering defect passivation and crystallization. The suppressed trap-assisted nonradiative recombination due to the decreased defect density and enhanced carrier lifetime should be main reason for enhanced open-circuit voltage (*V*_OC_) and fill factor (FF).

### Interfacial Carrier Dynamics Investigation

**Fig. 4:**
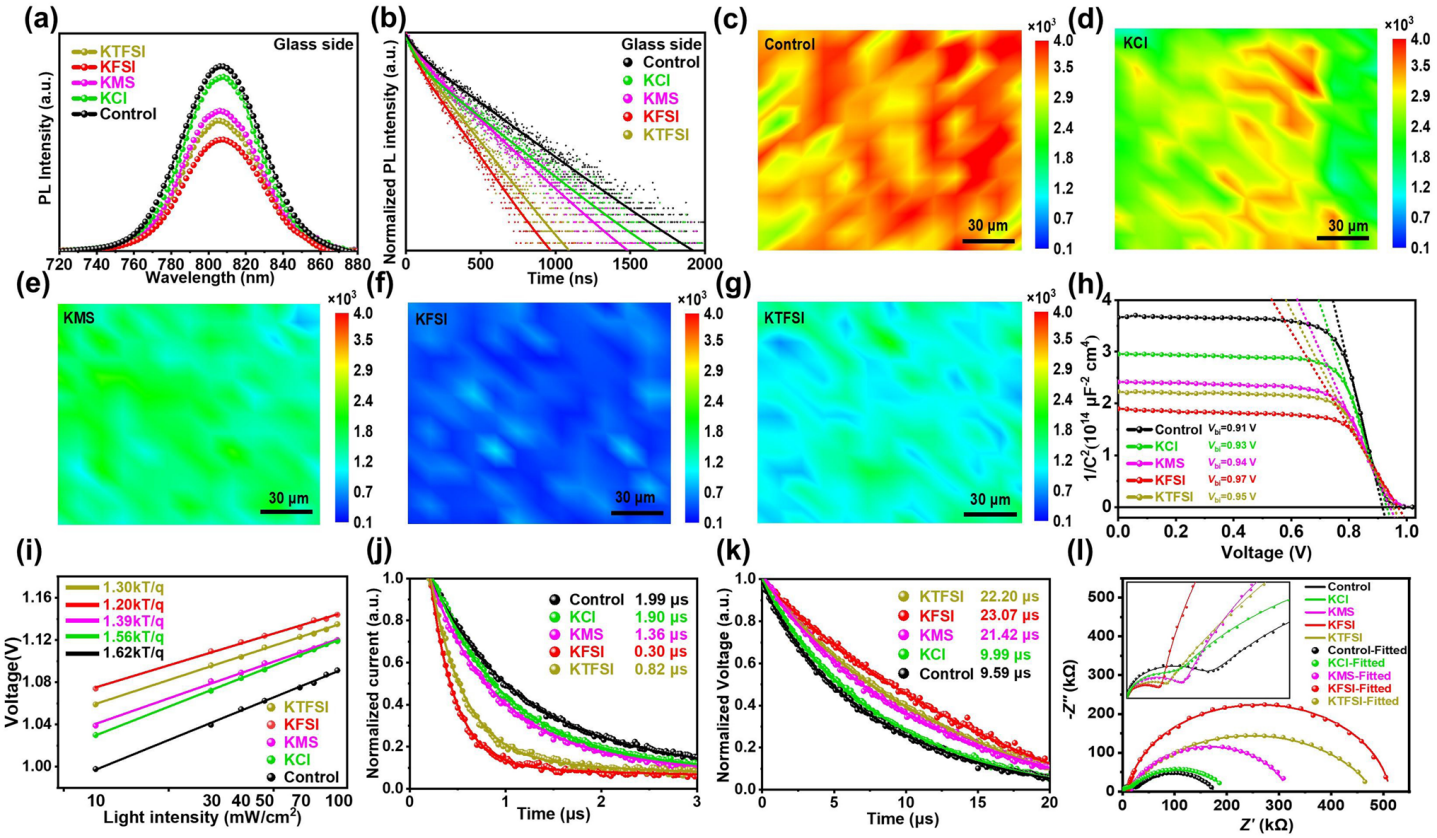
**a** SSPL and **b** TRPL spectra of the perovskite films deposited on SnO_2_ films without and with modifiers. PL mapping images of the perovskite films prepared on the **c** bare SnO_2_ and SnO_2_ modified by **d** KCl, **e** KMS, **f** KFSI and **g** KTFSI. **h** Mott–Schottky analysis, **i** TPC, **j** TPV and **k** *V*_OC_ as a function of light intensities for the control and modified PSCs. **l** Nyquist plots of the control and modified devices measured at a bias of 0.8 V in the frequency range of 1 MHz to 0.01 Hz in the dark. An enlarged view of the high-frequency region is shown in the inset

From Fig. 4a–b and Table S4, we observed gradually reduced PL intensities and carrier lifetimes in the order of SnO_2_/perovskite, SnO_2_/KCl/perovskite, SnO_2_/KMS/perovskite, SnO_2_/KTFSI/perovskite, and SnO_2_/KFSI/perovskite, demonstrating gradually improved charge extraction, which is because of ameliorated energy band alignment and inhibited trap-mediated nonradiative recombination. The PL mappings also certified ameliorated charge extraction, as presented in Fig. 4c–g. In Fig. 4h, in contrast to the control device, the KCl, KMS, KTFSI and KFSI based devices demonstrated increased built-in potential (*V*_bi_), which is suggestive of improved carrier separation and extraction due to more favorable band alignment. Subsequently, the transient photocurrent (TPC) decay in Fig. 4j also revealed gradually improved interfacial charge transfer and extraction in the order of the control (1.99 μs), KCl (1.90 μs), KMS (1.36 μs), KTFSI (0.82 μs), and KFSI (0.30 μs) based devices. The transient photovoltage (TPV) results as shown in Fig. 4k demonstrated that the carrier decay lifetime of the control device (9.59 μs) was increased to 9.99, 21.42, 22.20 and 23.07 μs for KCl, KMS, KTFSI, and KFSI modified devices, respectively, suggesting nonradiative recombination was suppressed owing to effective defect passivation, ameliorative band alignment and enhanced perovskite crystallization. The *V*_OC_ was plotted as a function of light intensity in Fig. 4i. The ideal factor (*n*) was calculated by Eq. (6) of fitting straight line:

6 $${\text{slope}} = {\text{nkT}}/q$$

where *k* stands for the Boltzmann constant, *T* represents the absolute temperature, and *q* denotes the elementary charge. Compared with the control device (1.62), the devices with KCl, KMS, KTFSI and KFSI realized lower *n* values of 1.56, 1.39, 1.30 and 1.20, respectively, which is indicative of memorably restrained non-radiative recombination, agreeing with the above TPV results. The exponential relationship between *J*_SC_ and light intensity is also shown in Fig. S17 and the exponential factor α of the KFSI modified device (0.979) was highest, followed by the device with KTFSI (0.968), the device with KMS (0.956), the device with KCl (0.950), and the control device (0.948). This indicates that KFSI modified device showed efficient carrier transport and extraction.

Electrochemical impedance spectroscopy (EIS) measurement was performed to explore the underlying mechanism of charge transfer and recombination in PSCs (Figs. 4l and S18). The decrease in charge transfer resistance (*R*_ct_) values in the high frequency region and the increase in carrier recombination resistance (*R*_rec_) in the low frequency region indicate that the nonradiative recombination was suppressed and interfacial charge transfer was promoted after interface modification. The improved interfacial energy band alignment mainly contributed to ameliorated interfacial carrier extraction and transfer. The reduced interfacial defects, reduced grain boundary density, increased crystallinity and improved crystal plane orientation should be primarily responsible for inhibited carrier nonradiative recombination.

### Photovoltaic Performance

**Fig. 5:**
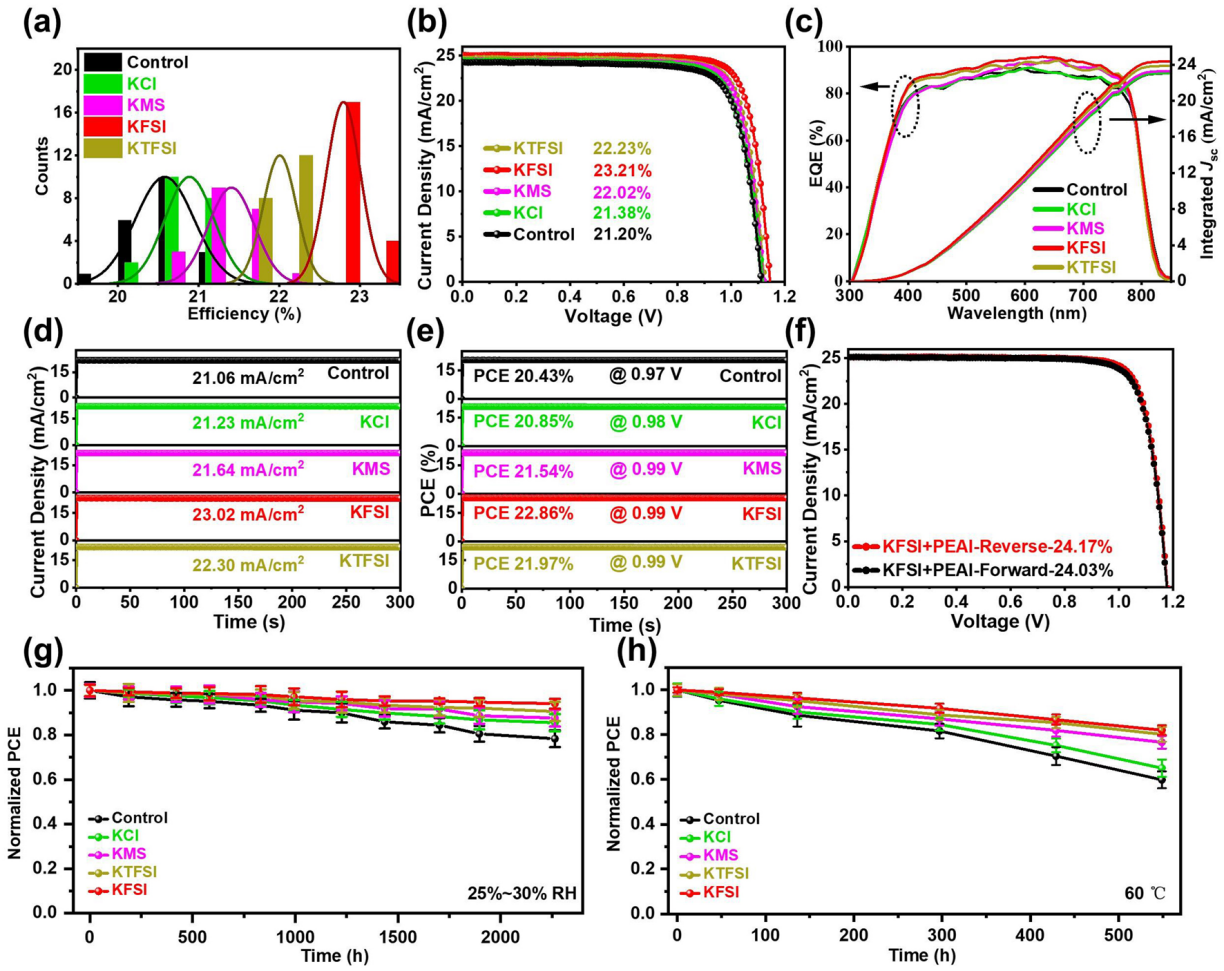
**a** Statistical distribution of the PCEs of the PSCs based on the SnO_2_ films without and with KCl, KMS, KTFSI and KFSI. The statistical data were obtained from 20 individual cells for each kind of device. **b** *J–V* curves and **c** EQE spectra of the best-performing devices based on bare SnO_2_ film and KCl, KMS, KTFSI and KFSI modified SnO_2_ films. *J–V* curves were measured under simulated AM 1.5G one sun illumination of 100 mW cm^−2^. **d** Steady-state current density and **e** PCE versus time for the best-performing devices employing pristine and modified SnO_2_ films measured at the maximum power point. **f** *J-V* curves of the KFSI-modified champion PSCs with PEAI posttreatment. PCE evolution of the unencapsulated control and modified **g** under 25%-30% relative humidity at room temperature in the dark, **h** at 60 °C in the dark in the nitrogen-filled glovebox. Error bars represent the standard deviation of five devices

The photovoltaic performance of the planar PSCs modified by various concentrations of modifiers based on two step method were compared (Figs. S19–S22 and Tables S5–S8). Obviously, it can be found in Figs. S22 and 5a that increased PCE was ascribed to simultaneous enhancement of *V*_OC_, short-circuit current density (*J*_SC_) and FF. Improved *V*_OC_ and FF should be put down to reduced defect density of perovskite layer and ETL, increased carrier lifetimes, ameliorated perovskite crystallization and suppressed interfacial nonradiative recombination. Among all modifiers, KFSI-modified device exhibited the best photovoltaic performance. All potassium salts containing non-halogen anions are better in device performance with respect to KCl and reference device. This indicates that non-halogen anions could be very promising in defect passivation and crystallization enhancement than halide anions in consideration of structure tunability of the former. Figure 5b exhibits *J-V* curves of the best-performing PSCs without and with KCl, KMS, KFSI and KTFSI. Compared with the control device (a *J*_SC_ of 24.50 mA cm^−2^, a *V*_OC_ of 1.114 V, an FF of 0.789 and PCE of 21.20%), the KCl-modified device showed a slightly enhanced PCE of 21.38% (a *J*_SC_ of 24.58 mA cm^−2^, a *V*_OC_ of 1.119 V, and an FF of 0.790). In contrast, the devices based on potassium salts with non-halogen anions exhibited obvious PCE enhancement. The KMS, KTFSI and KFSI modified devices achieved a PCE of 22.02% (a *J*_SC_ of 24.68 mA cm^−2^, a *V*_OC_ of 1.121 V, and an FF of 0.804), a PCE of 22.23% (a *J*_SC_ of 24.77 mA cm^−2^, a *V*_OC_ of 1.139 V, and an FF of 0.809), and a PCE of 23.21% (a *J*_SC_ of 25.12 mA cm^−2^, a *V*_OC_ of 1.148 V, and an FF of 0.817), respectively. This suggests that it is of critical importance to incorporate F and S=O functional groups to enhance chemical interaction between non-halogen anions and interface. As illustrated in Fig. 5c, the integrated current densities of the control, KCl, KMS, KTFSI and KFSI modified devices were 23.03, 23.07, 23.30, 23.82 and 24.34 mA cm^−2^, respectively. It is revealed in Fig. 5d–e that steady-state current densities and PCEs of the control, KCl, KMS, KTFSI and KFSI modified devices after 300 s were 21.06 mA cm^−2^ and 20.43%, 21.23 mA cm^−2^ and 20.85%, 21.64 mA cm^−2^ and 21.54%, 22.30 mA cm^−2^ and 21.97%, and 23.02 mA cm^−2^ and 22.86%, respectively. The KFSI-modified PSC with PEAI post treatment delivered a further improved PCE of 24.17% in reverse scan and 24.04% in forward scan (Fig. 5f and Table S9).

Finally, we investigated the stability of the unencapsulated control and modified devices. Figure 5g shows the moisture stability of the control and modified devices under a relative humidity of 30% − 35% at 25 °C. After 2264 h, the control device degraded to 78% of its initial value whereas the KCl, KMS, KTFSI and KFSI-modified devices degraded to 86%, 88%, 91% and 94% of their initial PCEs, respectively, which is indicative of improved moisture stability after interface modification. The thermal stability was evaluated by aging unencapsulated devices at 60 °C in a nitrogen-filled glove box (Fig. 5h). After aging for 549 h, it was found that the KCl, KMS, KTFSI and KFSI-modified devices maintained 65%, 77%, 80% and 82% of their initial PCEs while only 60% for the control device, respectively, indicating that thermal stability was improved after interface modification. Figure 5i exhibit the photostability of the control and modified devices under one sun illumination at room temperature, where the devices were located in the glovebox filled with nitrogen. After 343 h, the control device degraded to 66% of its initial value whereas the KCl, KMS, KTFSI and KFSI-modified devices degraded to 72%, 74.7%, 78% and 85% of their initial PCEs, respectively, which is indicative of improved photostability after interface modification (Fig. S24).

The trap-assisted nonradiative recombination is one of main reasons for device degradation [52]. Therefore, the effective passivation of modifiers for the defects from perovskite and SnO_2_ films should be mainly responsible for improved device stability. In addition, improved interfacial contact by chemically bridging perovskite layer and ETL should be another important origin of enhanced stability [17, 27, 28]. In short, our interfacial modification strategy can enhance efficiency and stability simultaneously.

## Conclusions

In summary, we developed a buried interface stabilization strategy based on synergistic engineering of fluorine and sulfonyl functional groups. A series of potassium salts (KCl, KMS, KFSI and KTFSI) were used to modify SnO_2_/perovskite buried interface. Multiple chemical bonds were realized by simultaneous introduction of F and S=O. First, F can form hydrogen bond with organic cations and form coordination bond with Pb^2+^ and Sn^4+^. Second, S=O functional group and non-halogen anion can form coordination bond with Pb^2+^ and Sn^4+^. Finally, K^+^ and non-halogen anions can form ionic bonds with cation and halide vacancy defects, respectively. The crystallization kinetics was regulated through the compromise between chemical interaction strength and wettability of substrates. The introduction of F would enhance chemical interaction while it also would increase hydrophobicity of surface of ETL. Appropriate hydrophobicity is conducive to suppressing heterogeneous nucleation and thus promoting perovskite crystallization but too high hydrophobicity hinders perovskite crystallization. The KFSI-modified device obtained an appealing PCE of 24.17% along with excellent stability. This work demonstrated great potentials of non-halogen anions and provide a feasible and effective approach toward managing interfacial carrier via the synergistic design of organic functional groups.

### Supplementary Information

Below is the link to the electronic supplementary material.

Supplementary file1 (PDF 1852 KB)
