# Supplementary material for: Stabilizing Buried Interface via Synergistic Effect of Fluorine and Sulfonyl Functional Groups Toward Efficient and Stable Perovskite Solar Cells
Source: Nanomicro Lett. 2022 Dec 29;15:17. doi: 10.1007/s40820-022-00992-5 (PMC9800669; doi:10.1007/s40820-022-00992-5)
Supplement: Supplementary file 1 — Supplementary file1 (PDF 1852 KB) [file 40820_2022_992_MOESM1_ESM.pdf]

Supporting Information for

Stabilizing Buried Interface via Synergistic Effect of Fluorine and Sulfonyl Functional Groups toward Efficient and Stable Perovskite Solar Cells

Cheng Gong[1], Cong Zhang[1], Qixin Zhuang[1], Haiyun Li[1], Hua Yang[2], Jiangzhao Chen[1,]*, and Zhigang Zang[1,]*

[1]Key Laboratory of Optoelectronic Technology & Systems (Ministry of Education), Chongqing University, Chongqing 400044, P. R. China

[2]Institute of High Energy Physics, Chinese Academy of Sciences (CAS), Beijing 100049, P. R. China

*Corresponding authors. E-mails: jiangzhaochen@cqu.edu.cn (Jiangzhao Chen), zangzg@cqu.edu.cn (Zhigang Zang)

Supplementary Figures and Tables

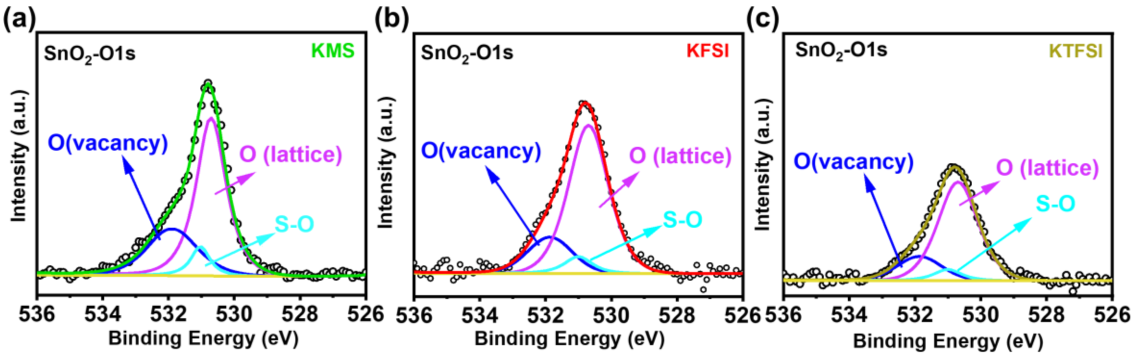

Fig. S1 O 1s XPS spectra of the SnO_2 with KMS, KFSI and KTFSI modification

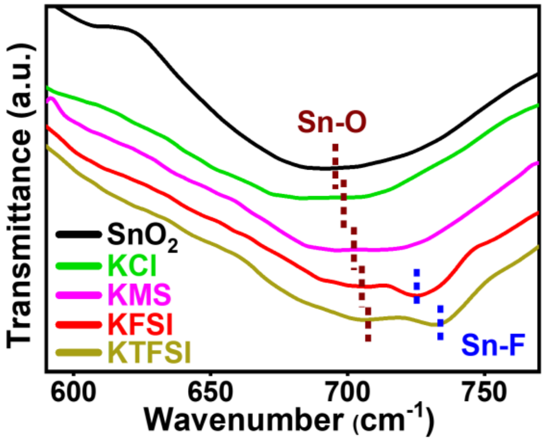

Fig. S2 FTIR spectra of the SnO_2 films without and with KCl, KMS, KTFSI and KFSI

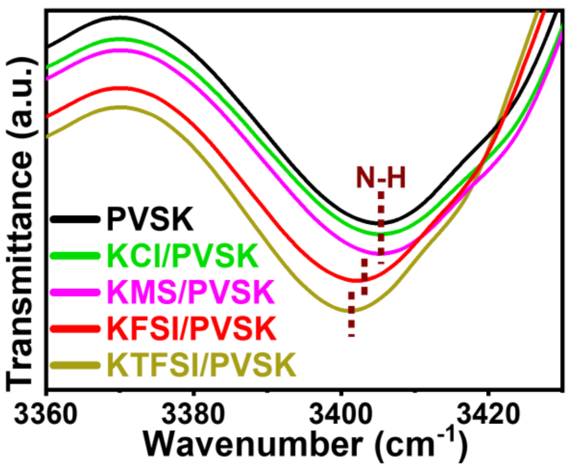

Fig. S3 FTIR spectra of the perovskite films without or with KCl, KMS, KTFSI and KFSI

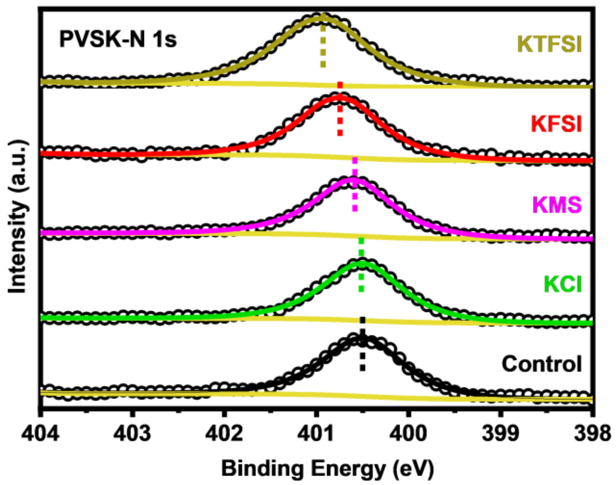

Fig. S4 N 1s XPS spectra of the perovskite films without or with KCl, KMS, KFSI and KTFSI modification. PVSK stands for perovskite

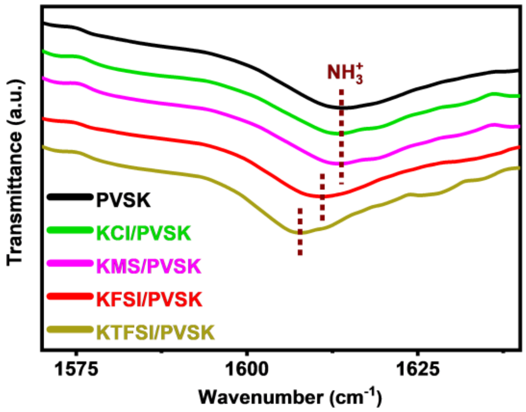

Fig. S5 FTIR spectra of the perovskite films with or without KCl, KMS, KFSI and KTFSI

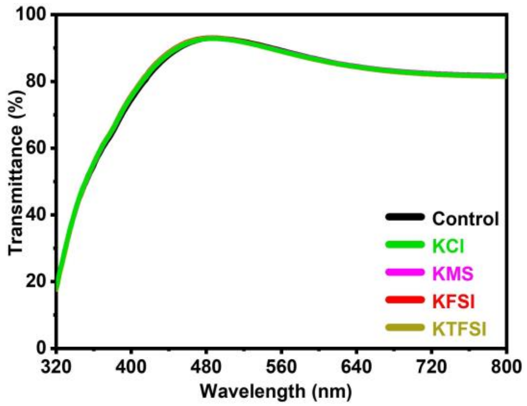

Fig. S6 Optical transmission spectra of the SnO_2 films without and with KCl, KMS, KFSI and KTFSI prepared on ITO substrates

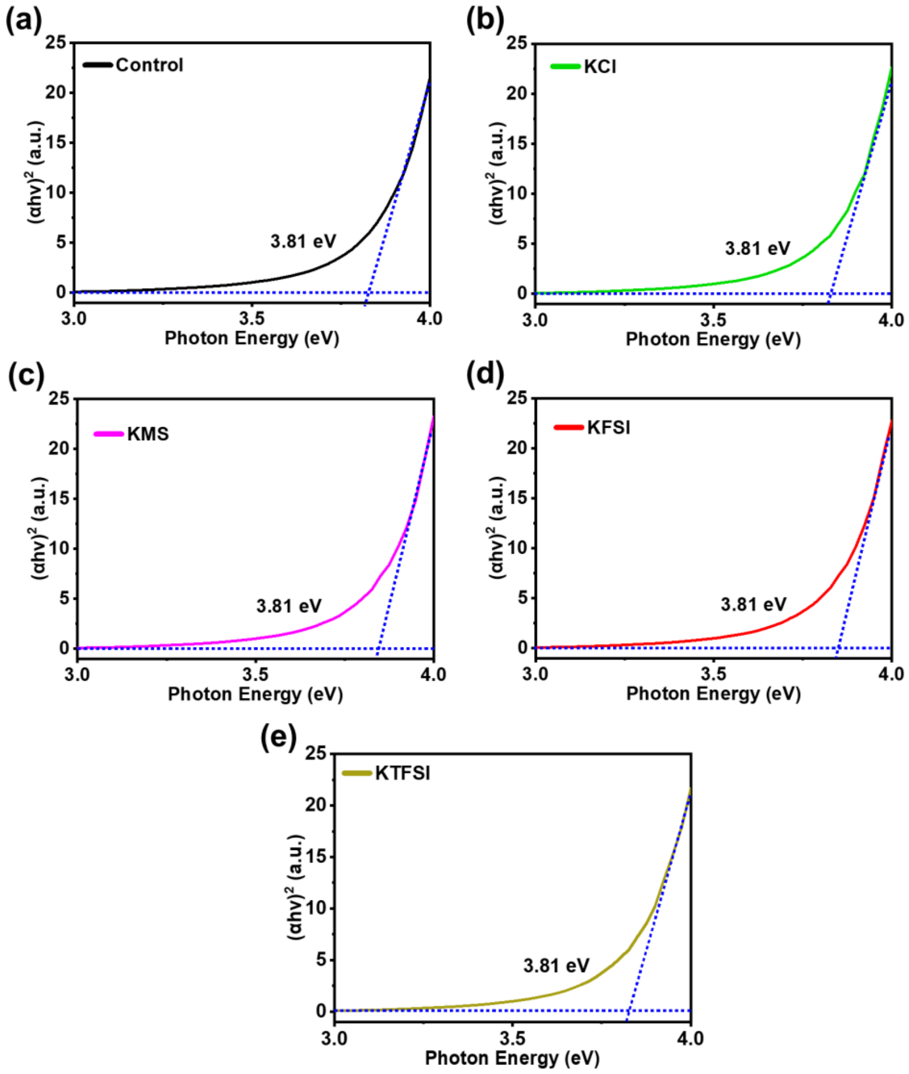

Fig. S7 The tauc plots of the SnO_2 films without and with KCl, KMS, KFSI or KTFSI modification

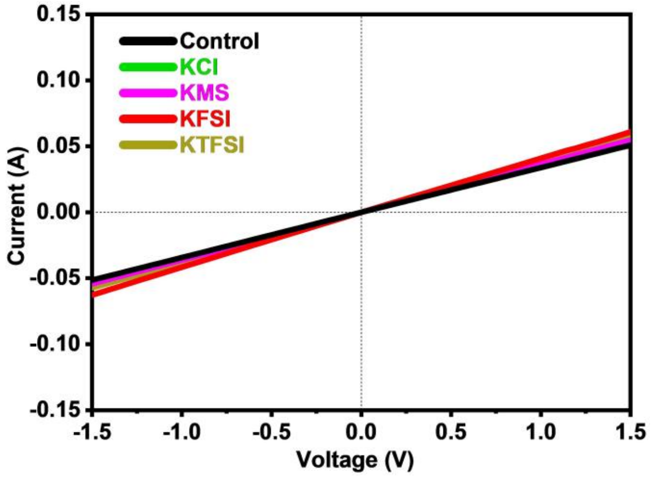

Fig. S8 *I*–*V* curves of ITO/SnO_2 without and with KCl, KMS, KFSI or KTFSI/Ag

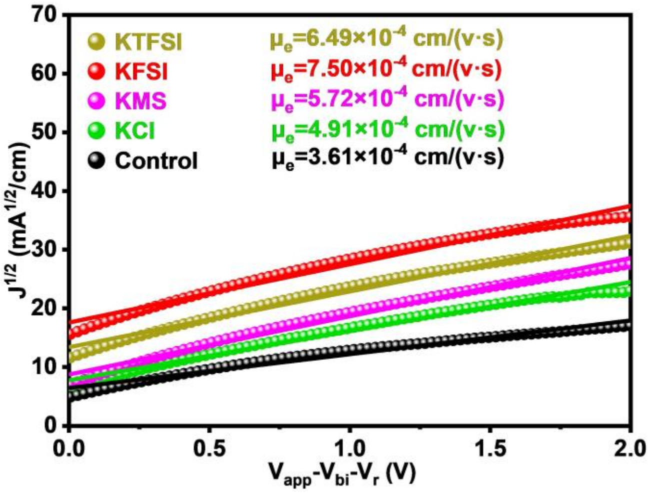

Fig. S9 Measurement of electron mobility of SnO_2, SnO_2/KCl, SnO_2/KMS, SnO_2/KFSI and SnO_2/KTFSI films by using SCLC model with ITO/PCBM/ETL/PCBM/Ag device structure

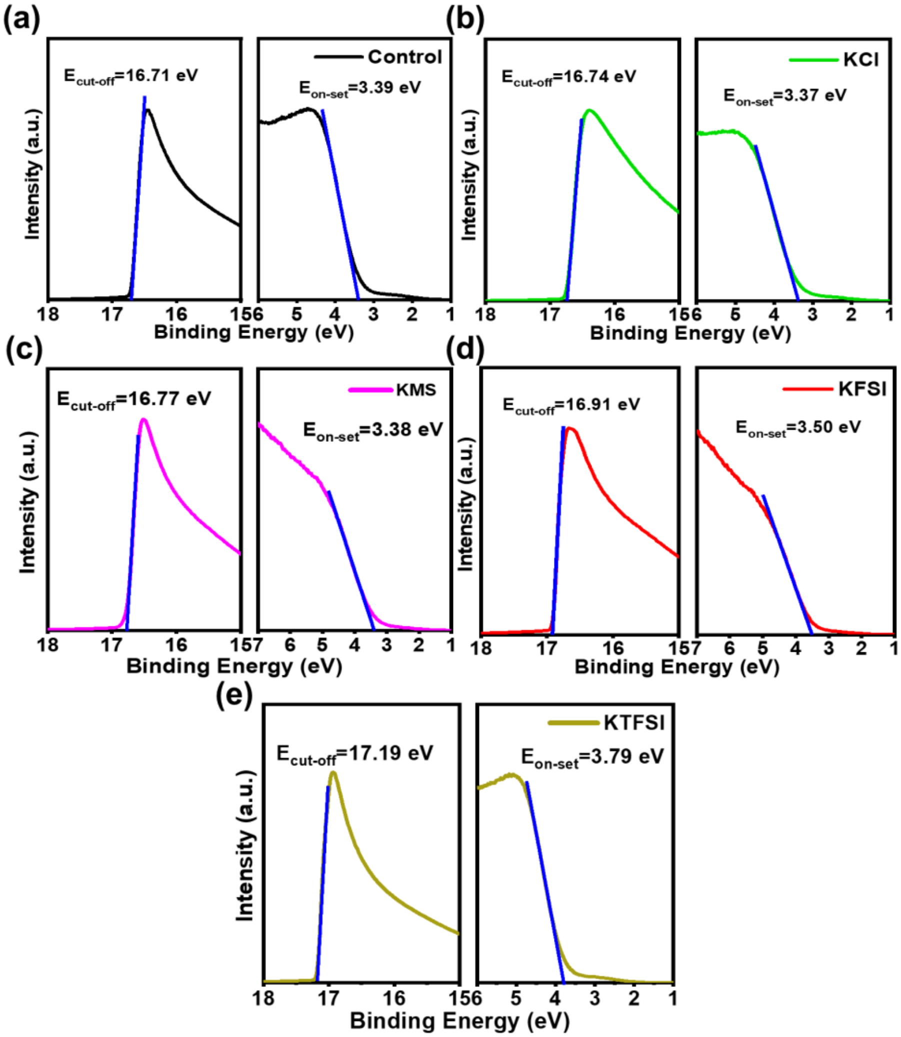

Fig. S10 UPS spectra, on-set energy $E_{on\text{-}set}$ and cut-off energy ($E_{cut\text{-}off}$) of the (**a**) bare SnO_2, (**b**) SnO_2/KCl, (**c**) SnO_2/KMS, (**d**) SnO_2/KFSI and (**e**) SnO_2/KTFSI

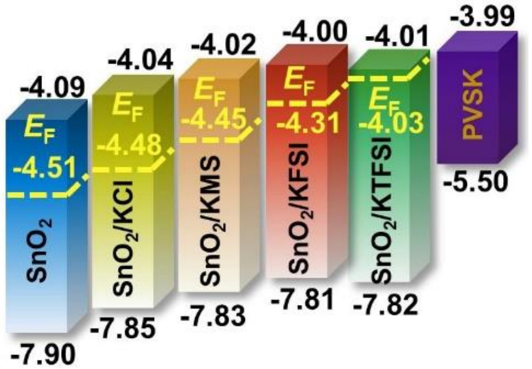

Fig. S11 Energy level diagram of the SnO_2 films without and with modifiers as well as perovskite film

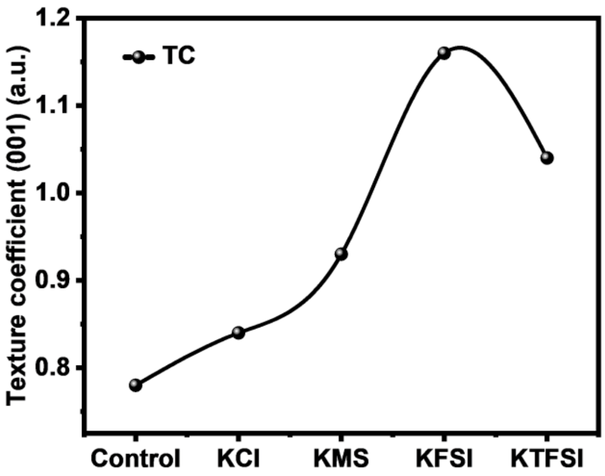

Fig. S12 The texture coefficients in for the (001) planes of perovskite films with or without KCl, KMS, KFSI and KTFSI

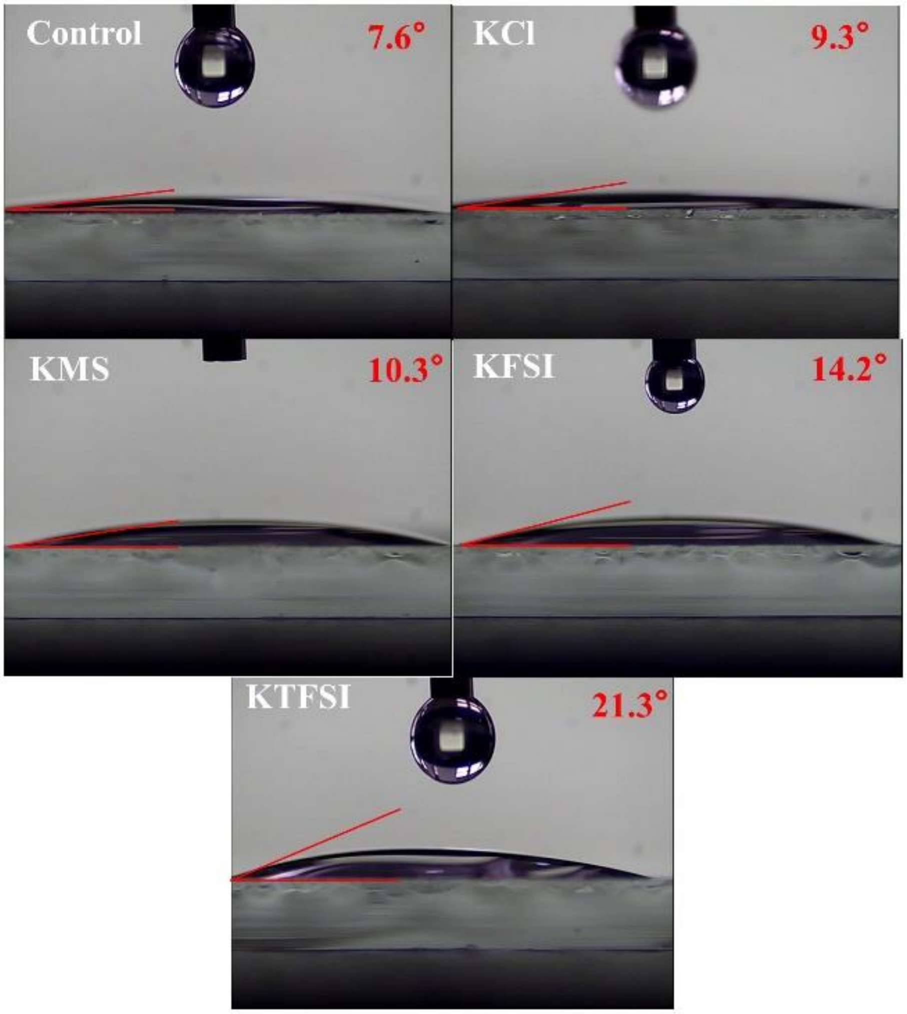

Fig. S13 Contact angle measurement of the DMF/DMSO (9:1) solution droplet on pristine and modified SnO_2 films with KCl, KMS, KFSI and KTFSI

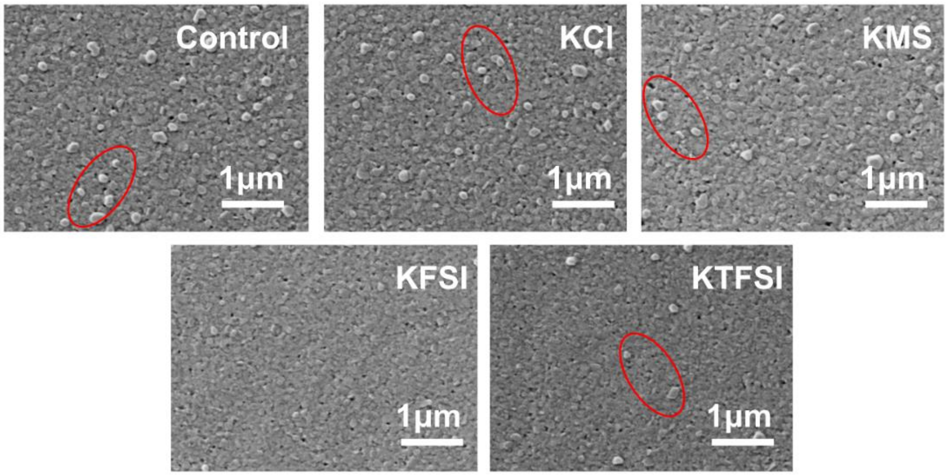

Fig. S14 Top-view SEM images of the PbI_2 films prepared on SnO_2 without and with KCl, KMS, KFSI and KTFSI

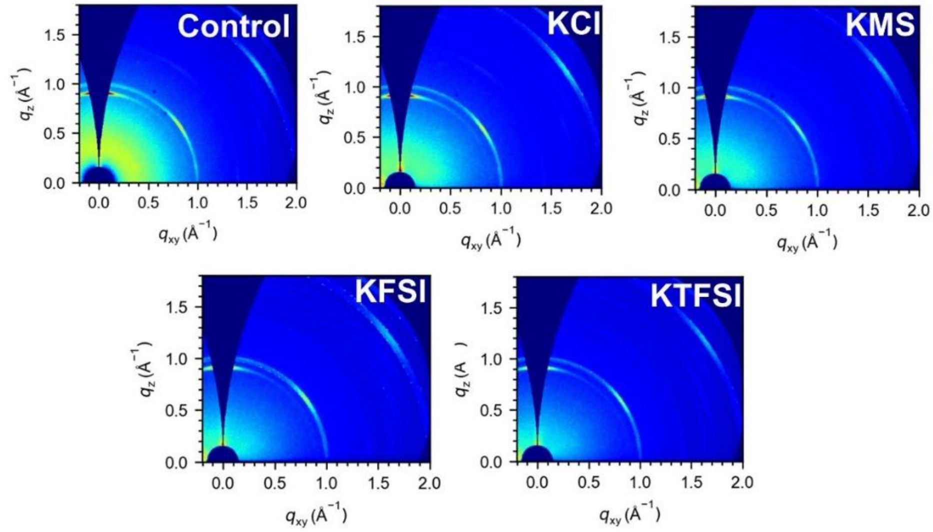

Fig. S15 GIWAXS patterns of the perovskite films spin-coated on the bare and modified SnO_2 films

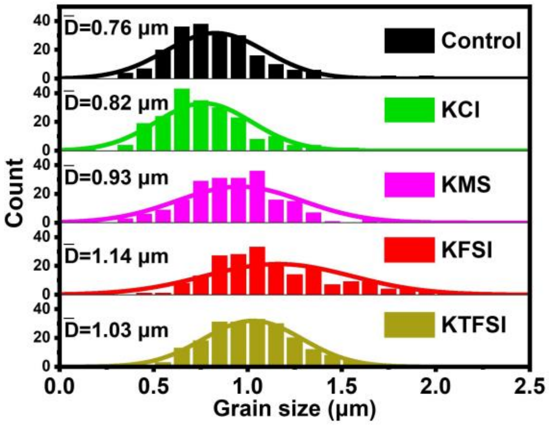

Fig. S16 The grain size statistics from the SEM images in Fig. 2a

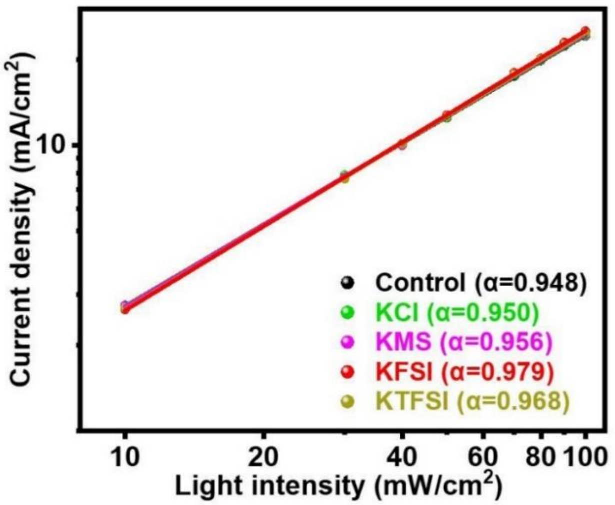

Fig. S17 J_{SC} dependent on various light intensities

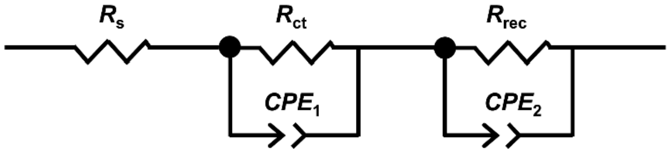

Fig. S18 The equivalent circuit model for EIS fitting. The equivalent circuit consisted of the series resistance (R_s), the transfer resistance R_{ct}, and the recombination resistance R_{rec}

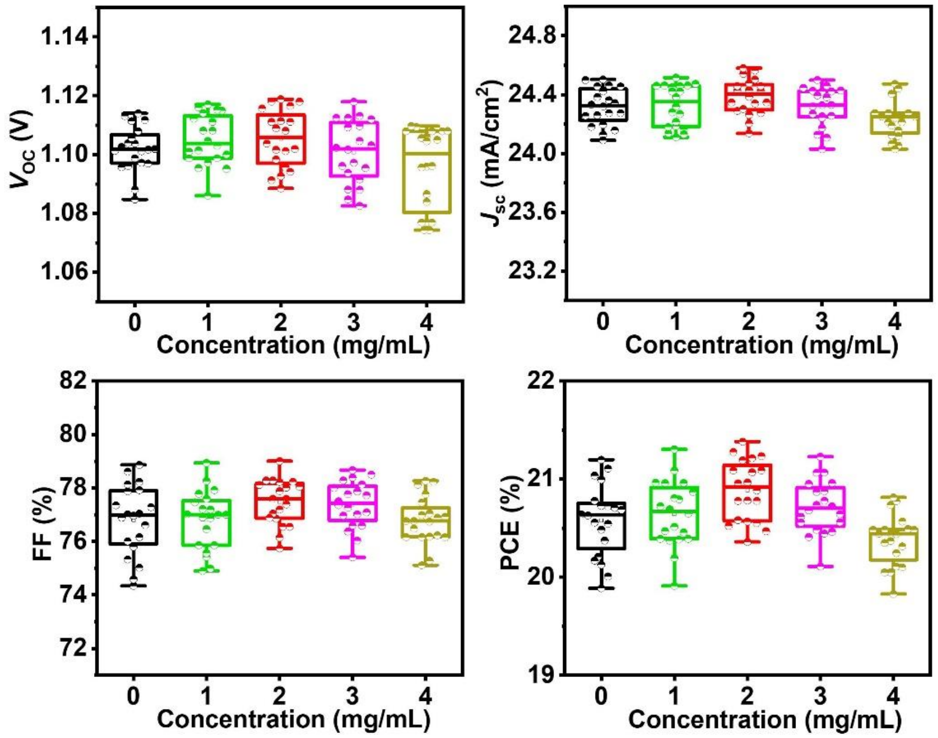

Fig. S19 Statistical distribution diagram of the photovoltaic parameters of the PSCs prepared based on various concentrations of KCl. The statistical data were obtained from 20 individual cells for each kind of device

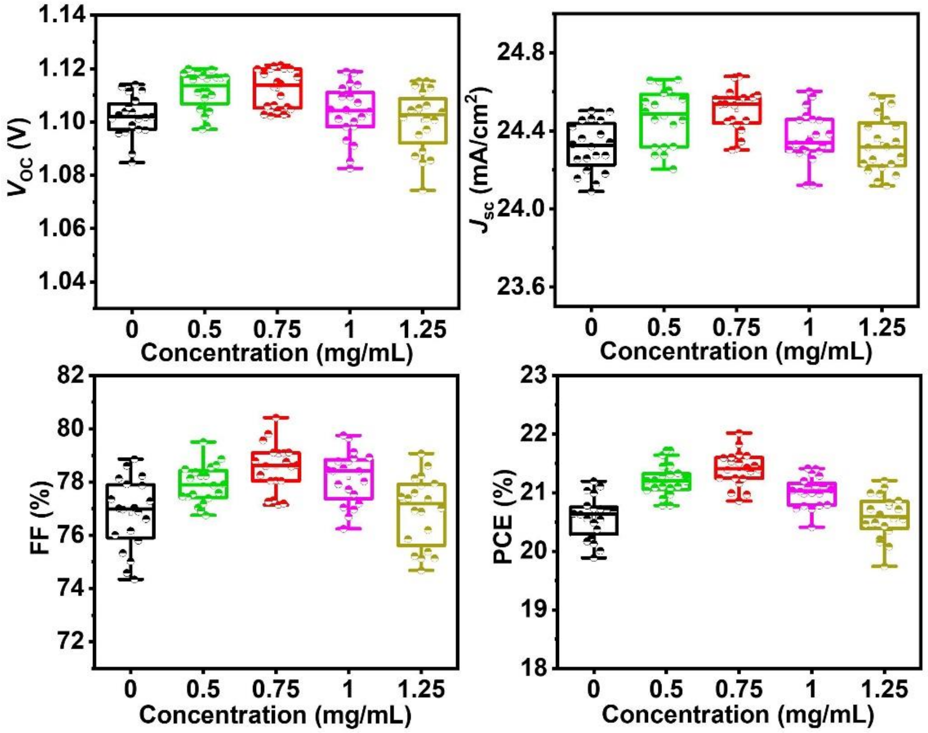

Fig. S20 Statistical distribution diagram of the photovoltaic parameters of the PSCs modified by various concentrations of KMS. The statistical data were obtained from 20 individual cells for each kind of device

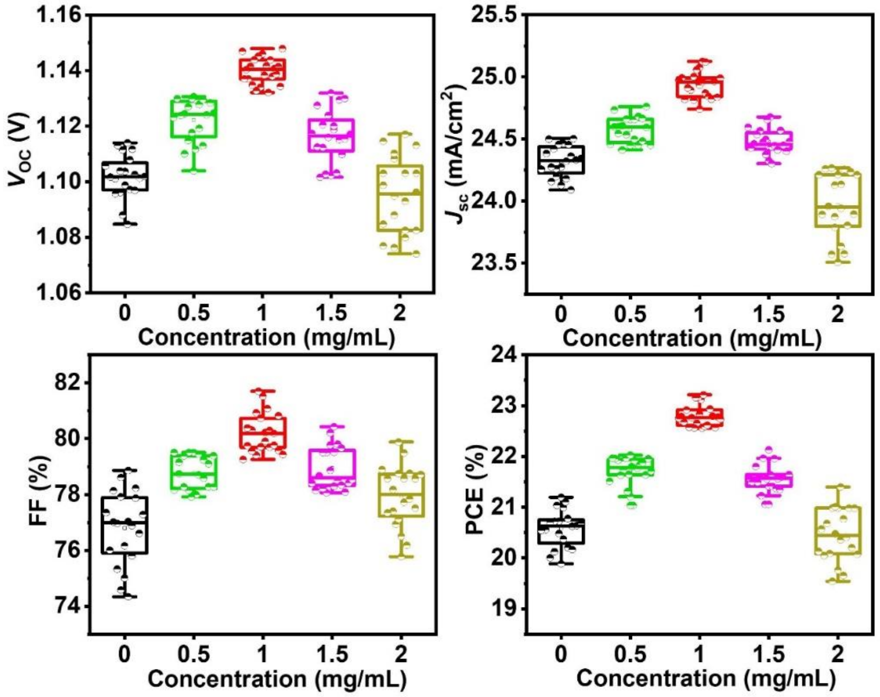

Fig. S21 Statistical distribution diagram of the photovoltaic parameters of the PSCs modified by various concentrations of KFSI. The statistical data were obtained from 20 individual cells for each kind of device

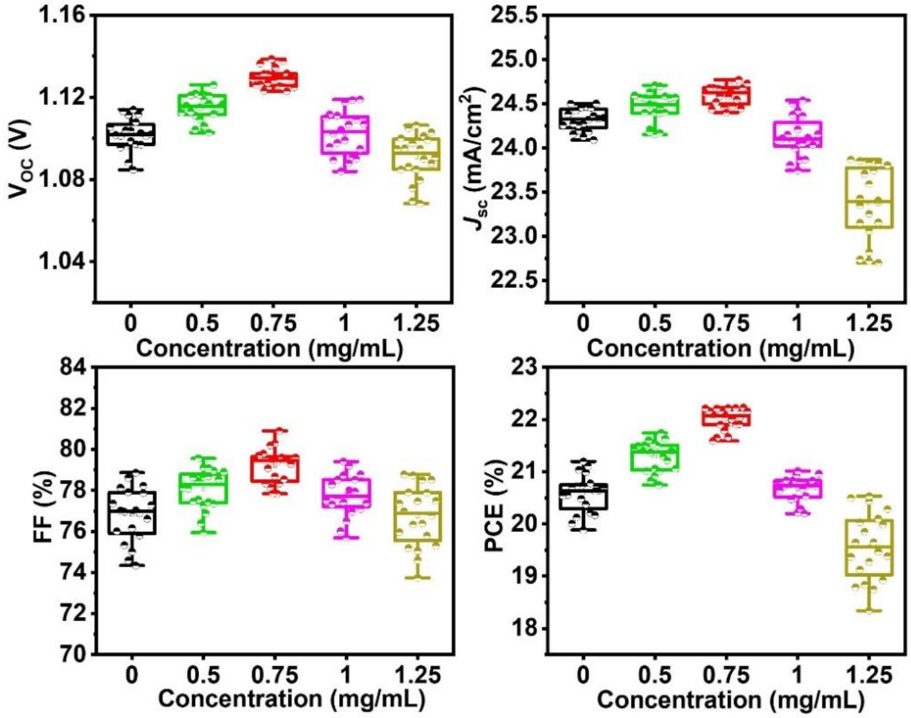

Fig. S22 Statistical distribution diagram of the photovoltaic parameters of the PSCs prepared based on various concentrations of KTFSI. The statistical data were obtained from 20 individual cells for each kind of device

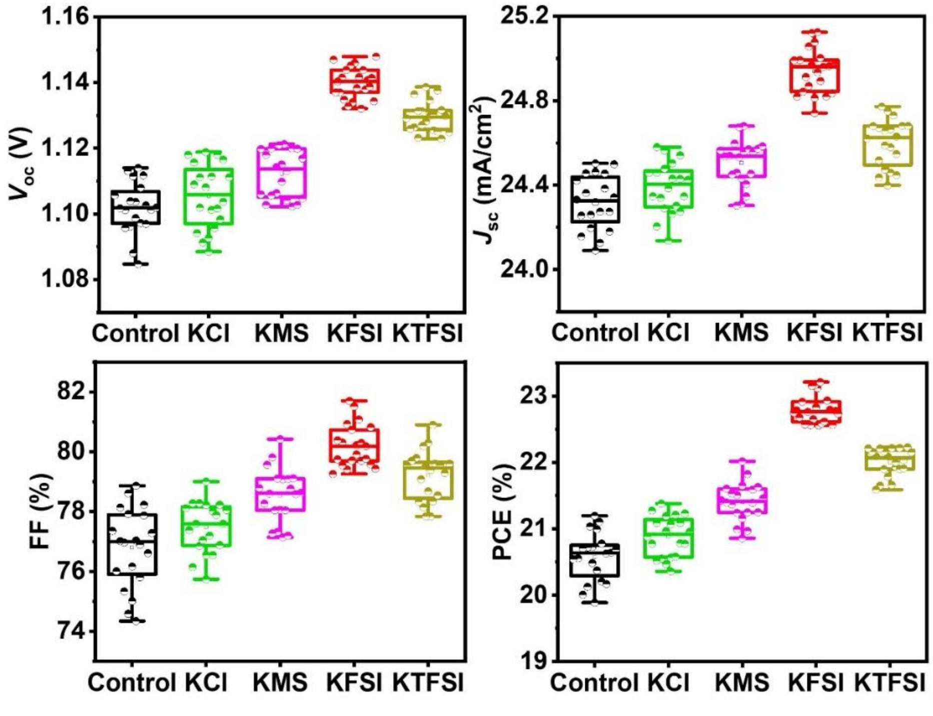

Fig. S23 Statistical distribution diagram of the photovoltaic parameters of the control and modified PSCs prepared based on the optimum concentration of KCl, KMS, KFSI and KTFSI. The statistical data were obtained from 20 individual cells for each kind of device

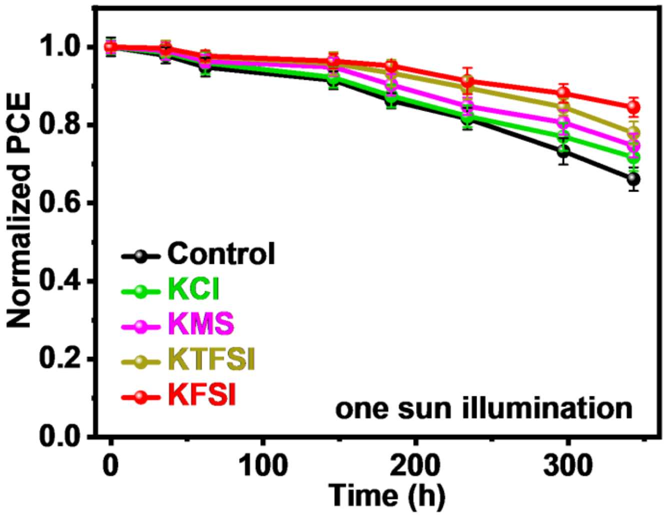

Fig. S24 PCE evolution of the unencapsulated control and modified under one sun illumination of 100 mW/cm^2 provided by white light LED at room temperature. Error bars represent the standard deviation of five devices

Table S1 Calculated valence band (E_{VB}) and conduction band (E_{CB}) from $E_{cut\text{-}off}$, E_F and E_g for the SnO_2 films without and with KCl, KMS, KFSI, and KTFSI

Films	$E_{cut\text{-}off}$ (eV)	$E_{on\text{-}set}$ (eV)	E_F (eV)	E_{VB} (eV)	E_g (eV)	E_{CB} (eV)
SnO_2	16.71	3.39	-4.51	-7.90	3.81	-4.09
SnO_2/KCl	16.74	3.37	-4.48	-7.85	3.81	-4.04
SnO_2/KMS	16.77	3.38	-4.45	-7.83	3.81	-4.02
SnO_2/KFSI	16.91	3.50	-4.31	-7.81	3.81	-4.00
SnO_2/KTFSI	17.19	3.79	-4.03	-7.82	3.81	-4.01

Table S2 The calculated texture coefficient (*TC*) for different films.

Samples	Control	KCl	KMS	KFSI	KTFSI
Texture Coefficient (001)	0.78	0.84	0.93	1.16	1.04

Table S3 Fitted results of TRPL curves of the perovskite films deposited on glass with or without modifiers. The PVSK represents perovskite layer

	Samples	Glass/ PVSK	Glass/KCl/ PVSK	Glass/KMS/ PVSK	Glass/KFSI/ PVSK	Glass/KTFSI/ PVSK
Glass side	A_1(%)	0.32	0.28	0.35	0.12	0.37
	τ_1(ns)	54.04	79.45	77.39	83.81	82.71
	A_2(%)	0.68	0.72	0.65	0.88	0.63
	τ_2(ns)	465.5	539.38	585.73	639.89	657.24
	τ_{ave}(ns)	444.18	514.46	551.97	630.13	617.70
PVSK side	A_1(%)	0.42	0.29	0.37	0.43	0.37
	τ_1(ns)	44.84	50.48	65.93	58.2	59.29
	A_2(%)	0.58	0.71	0.63	0.57	0.63
	τ_2(ns)	276.67	312.58	386.48	496.89	406.88
	τ_{ave}(ns)	252.32	296.36	357.29	461.27	379.48

Table S4 Fitted results of TRPL curves of the perovskite films deposited on SnO_2 without or with modifiers. The PVSK represents the perovskite layer

Samples	A_1(%)	τ_1(ns)	A_2(%)	τ_2(ns)	τ_{ave}(ns)
SnO_2/PVSK	0.28	69.79	0.72	378.93	358.27
SnO_2/KCl/PVSK	0.37	52.05	0.63	341.78	317.99
SnO_2/KMS/PVSK	0.34	75.41	0.66	295.36	269.79
SnO_2/KFSI/PVSK	0.34	39.2	0.66	190.49	175.99
SnO_2/KTFSI/PVSK	0.26	53.94	0.74	214.04	201.02

Table S5 Photovoltaic parameters of the PSCs based on SnO_2 films modified by different concentrations of KCl in the range of 0 to 5 mg/mL. *J-V* curves were measured with a scan rate of 100 mV/s under AM 1.5G illumination

Devices		J_{SC} (mA/cm^2)	V_{OC} (V)	FF	PCE (%)
Control	Average	24.32±0.13	1.102±0.008	0.768±0.013	20.58±0.36
	Champion	24.50	1.114	0.789	21.20
1 mg/mL	Average	24.32±0.13	1.105±0.008	0.768±0.011	20.64±0.34
	Champion	24.52	1.117	0.789	21.30

2 mg/mL	Average	24.39±0.12	1.105±0.008	0.775±0.008	20.89±0.31
	Champion	24.58	1.119	0.790	21.38
3 mg/mL	Average	24.32±0.13	1.101±0.010	0.774±0.009	20.71±0.32
	Champion	24.50	1.118	0.787	21.23
4 mg/mL	Average	24.23±0.13	1.096±0.014	0.768±0.009	20.38±0.32
	Champion	24.47	1.110	0.783	20.81

Table S6 Photovoltaic parameters of the PSCs based on SnO_2 films modified by different concentrations of KMS in the range of 0 to 1.25 mg/mL. *J-V* curves were measured with a scan rate of 100 mV/s under AM 1.5G illumination

Devices		J_{SC} (mA/cm^2)	V_{OC} (V)	FF	PCE (%)
Control	Average	24.32±0.13	1.102±0.008	0.768±0.013	20.58±0.36
	Champion	24.50	1.114	0.789	21.20
0.5 mg/mL	Average	24.47±0.14	1.112±0.007	0.779±0.007	21.20±0.25
	Champion	24.66	1.120	0.795	21.72
0.75 mg/mL	Average	24.51±0.11	1.112±0.007	0.785±0.007	21.41±0.25
	Champion	24.68	1.121	0.804	22.02
1 mg/mL	Average	24.34±0.13	1.103±0.010	0.782±0.010	21.01±0.26
	Champion	24.60	1.119	0.797	21.41
1.25 mg/mL	Average	24.33±0.14	1.100±0.011	0.769±0.013	20.59±0.37
	Champion	24.58	1.115	0.791	21.21

Table S7 Photovoltaic parameters of the PSCs based on SnO_2 films modified by different concentrations of KFSI in the range of 0 to 2 mg/mL. *J-V* curves were measured with a scan rate of 100 mV/s under AM 1.5G illumination

Devices		J_{SC} (mA/cm^2)	V_{OC} (V)	FF	PCE (%)
Control	Average	24.32±0.13	1.102±0.008	0.768±0.013	20.58±0.36
	Champion	24.50	1.114	0.789	21.20
0.5 mg/mL	Average	24.57±0.10	1.122±0.008	0.788±0.006	21.72±0.27
	Champion	24.76	1.131	0.795	22.02
1 mg/mL	Average	24.94±0.11	1.140±0.004	0.802±0.007	22.80±0.21
	Champion	25.12	1.148	0.817	23.21
1.5 mg/mL	Average	24.48±0.11	1.116±0.009	0.789±0.008	21.57±0.25
	Champion	24.68	1.132	0.804	22.13
2 mg/mL	Average	23.97±0.26	1.095±0.013	0.779±0.011	20.46±0.52
	Champion	24.27	1.117	0.799	21.40

Table S8 Photovoltaic parameters of the PSCs based on SnO_2 film modified by different concentrations of KTFSI in the range of 0 to 1.25 mg/mL. *J-V* curves were measured with a scan rate of 100 mV/s under AM 1.5G illumination

Devices		J_{SC} (mA/cm^2)	V_{OC} (V)	FF	PCE (%)
Control	Average	24.32±0.13	1.102±0.008	0.768±0.013	20.58±0.36
	Champion	24.50	1.114	0.789	21.20
0.5 mg/mL	Average	24.46±0.13	1.115±0.006	0.781±0.010	21.29±0.29
	Champion	24.71	1.126	0.796	21.74
0.75 mg/mL	Average	24.60±0.11	1.130±0.005	0.792±0.008	22.01±0.21
	Champion	24.77	1.139	0.809	22.23
1 mg/mL	Average	24.13±0.22	1.102±0.011	0.777±0.010	20.67±0.28
	Champion	24.54	1.119	0.794	21.01
1.25 mg/mL	Average	23.37±0.41	1.091±0.011	0.767±0.015	19.57±0.65
	Champion	23.87	1.106	0.788	20.53

Table S9 Photovoltaic parameters of the KFSI-modified best-performing PSCs with PEAI posttreatment. *J-V* curves were measured with a scan rate of 100 mV/s under AM 1.5G illumination

Sweep direction	J_{SC} (mA/cm^2)	V_{OC} (V)	FF	PCE (%)
Forward scan	25.12	1.177	0.813	24.04
Reverse scan	25.09	1.179	0.817	24.17
